# Fermentative production of vitamin B_12_ by *Propionibacterium shermanii* and *Pseudomonas denitrificans* and its promising health benefits: A review

**DOI:** 10.1002/fsn3.4428

**Published:** 2024-09-30

**Authors:** Anjali Tripathi, Vinay Kumar Pandey, Parmjit S. Panesar, Anam Taufeeq, Hridyanshi Mishra, Sarvesh Rustagi, Sumira Malik, Béla Kovács, Tejas Suthar, Ayaz Mukarram Shaikh

**Affiliations:** ^1^ Department of Biotechnology, Sharda School of Engineering and Technology Sharda University Greater Noida India; ^2^ Research and Development Cell, Biotechnology Department Manav Rachna International Institute of Research and Studies (Deemed to Be University) Faridabad Haryana India; ^3^ Department of Food Engineering and Technology Sant Longowal Institute of Engineering and Technology Longowal Punjab India; ^4^ Department of Biotechnology, Faculty of Engineering and Technology Rama University Kanpur Uttar Pradesh India; ^5^ Department of Biotechnology Axis Institute of Higher Education Kanpur Uttar Pradesh India; ^6^ Department of Food Technology, School of Applied and Life Sciences Uttaranchal University Dehradun Uttarakhand India; ^7^ Amity Institute of Biotechnology Amity University Jharkhand Ranchi India; ^8^ Department of Biotechnology University Center for Research & Development (UCRD) Chandigarh University Mohali Punjab India; ^9^ Faculty of Agriculture, Food Science and Environmental Management Institute of Food Science University of Debrecen Debrecen Hungary; ^10^ Independent Research Chicago Illinois USA

**Keywords:** cobalamin, fermentation, microbial production, vitamin B_12_

## Abstract

Cobalamin, generally known as vitamin B_12_, is a crucial component required for humans in several physiological processes. It has been produced from sources that are derived from animals, making it difficult for vegetarians and vegans to consume the recommended amount each day. The importance of vitamin B_12_ in red blood cell production, DNA synthesis, and brain processes has been highlighted. Recent studies have looked at different methods of producing vitamin B_12_, such as microbial fermentation. *Propionibacterium shermanii* and *Pseudomonas denitrificans* have demonstrated remarkable potential as fermented sources of vitamin B_12_. Compared to conventional sources, the bioavailability of vitamin B_12_ produced by *P. denitrificans* and *P. shermanii* is more effective in meeting dietary needs. Vitamin B_12_ can be produced naturally by *P. denitrificans*. It is equipped with the enzymes and metabolic pathways required to produce this vital vitamin. The fermentation of several dietary substrates by *P. shermanii* can improve nutrient bioavailability. *P. shermanii* generates enzymes during fermentation that aid in the breakdown of complex nutrients, facilitating easier absorption and utilization by the body. The motive of the following critical evaluation is to assess the advantages of vitamin B_12_ for health and the capacity of *P. denitrificans* and *P. shermanii* to produce it through fermentation.

## Graphical abstract

Vitamin B_12_ is essential to human physiological processes, it might be challenging for vegans and vegetarians to get enough of it in their diets. *Propionibacterium shermanii* and *Pseudomonas denitrificans* have been demonstrated as promising fermented sources of vitamin B_12_. Vitamin B_12_ is naturally produced by *P. denitrificans*, but *P. shermanii* uses enzymes to increase the bioavailability of nutrients.

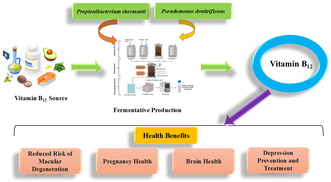

## 1 INTRODUCTION

Cobalamin, or vitamin B_12_, is an essential water‐soluble molecule in the metabolism of numerous organisms (Kokande et al., 2024). Vitamin B_12_ is a critical micronutrient that is involved in numerous physiological processes, such as the synthesis of DNA, the formation of red blood cells, and the maintenance of neurological health. B_12_ possesses an intricate composition and a sophisticated biosynthesis process, comprising more than 30 biotransformation stages. The biosynthetic pathway in question is restricted to specific microbes and archaea. Even though the taxa capable of vitamin B_12_ synthesis are not necessarily interrelated, mammals and thus humans lack the ability to produce it (Calvillo et al., 2022). Vitamin B_12_ has conventionally been obtained from animal products; however, a viable substitute has arisen in the form of fermentative production, which utilizes microorganisms such as *Propionibacterium shermanii*, *Rhodobacter capsulatus*, *Sinorhizobium meliloti*, *Salmonella typhimurium*, *Bacillus megaterium*, *Escherichia coli*, *Pseudomonas denitrificans*, and many more (Fang et al., 2017). Exploring the microbial strains responsible for the fermentative production of vitamin B12 presents an opportunity to develop manufacturing processes that are both sustainable and efficient. Sharma et al. (2024) suggested that *Propionibacterium shermanii* and *Pseudomonas denitrificans* are examples of microbes that possess the capability of producing vitamin B_12_ via regulated fermentation procedures. Table 1 represents the list of certain bacterial strains, their pathways, and their mechanism of action involved in the production of vitamin B_12_. This approach provides an environmentally sustainable, scalable, and cost‐effective substitute for conventional methods of extracting substances from animal products. It has the potential to mitigate vitamin B_12_ deficiencies in various dietary patterns. Microbial fermentation is a food production method that not only guarantees the bioavailability of vitamin B_12_ but also adheres to principles of ethics and sustainability (Gharibzahedi et al., 2023).

**TABLE 1:** Categories of different bacterial strains following various pathways, intermediate compounds, and the action mechanism.

Bacterial strain	Pathway followed	Intermediate compound	Mechanism of action	References
*Rhodospirillum rubrum*	Anaerobic pathway	Cobinamide	Anaerobic fermentation of succinate and cobalt ions	Goraya and Kaur (2023)
*Methanosarcina barkeri*	Anaerobic pathway	Cobalamin	Anaerobic fermentation of methanol	Wang et al. (2023)
*Rhodobacter sphaeroides*	Anaerobic pathway	Cobalt‐precorrin	Anaerobic fermentation of propionic acid and cobalt ions	Piwowarek et al. (2018)
*Lactobacillaceae reuteri*	Anaerobic pathway	Cobalt‐precorrin	Anaerobic fermentation of glycerol and cobalt ions	Kristjansdottir et al. (2019)
*Bacillus megaterium*	Anaerobic pathway	Cobalt‐precorrin	Anaerobic fermentation of glucose and cobalt ions	Xie et al. (2021)
*Pseudomonas denitrificans*	De novo pathway	Cobinamide	Aerobic fermentation of 5‐aminolevulinic acid (ALA)	Balabanova et al. (2021)
*Streptomyces griseus*	Anaerobic pathway	Cobinamide	Anaerobic fermentation of propionic acid and cobalt ions	Barbuto Ferraiuolo et al. (2021)
*Salmonella enterica*	De novo pathway	Cobalt‐precorrin	Aerobic fermentation of cobalt ions and glucose	Balabanova et al. (2021)
*Escherichia coli*	Salvage pathway	Cobinamide	Heterologous expression of vitamin B12 synthesis genes	Fang et al. (2018)
*Propionibacterium freudenreichii*	De novo pathway	Cobinamide	Anaerobic fermentation of propionic acid	Kumar et al. (2023)
*Salmonella typhimurium*	De novo pathway	Cobalt‐precorrin	Aerobic fermentation of cobalt ions and glucose	Zheng et al. (2020)
Streptomyces avermitilis	Anaerobic pathway	Cobinamide	Anaerobic fermentation of propionic acid and cobalt ions	Ranaei et al. (2020)

The capacity of the *Propionibacterium shermanii and Pseudomonas denitrificans* to produce vitamin B_12_ via a fermentative process has been acknowledged. The bacterium exhibits distinctive metabolic pathways that facilitate its efficient synthesis of cobalamin (Balabanova et al., 2021). *Propionibacterium shermanii* has the capability to produce vitamin B_12_ through cultivation on appropriate substrates, including propionic acid and other organic compounds, within a controlled fermentation environment. The utilization of fermentation as a method of vitamin B_12_ production presents numerous benefits in comparison to conventional extraction techniques, including enhanced scalability, cost‐efficiency, and diminished environmental footprint. Considerable research has been devoted to examining the capacity of this bacterium to produce cobalamin under suitable growth conditions and precursor conditions (Piwowarek et al., 2018). The incorporation of *Pseudomonas denitrificans* into the fermentative synthesis of vitamin B_12_ enhances the process's adaptability by permitting the use of distinct microbial strains to fulfill production criteria. The potential of these microorganisms to produce vitamin B_12_ through fermentation is substantial, as it could be utilized to treat nutritional deficiencies and improve human health (Calvillo et al., 2022). An insufficiency of vitamin B_12_ can result in neurological disorders, as it is vital for the normal operation of the nervous system. Fermentation of vitamin B_12_ could provide a sustainable and dependable source of the vitamin, which could substantially aid in the fight against deficiency‐related health problems, particularly in populations that have limited access to animal‐derived foods. Individuals adhering to vegetarian or vegan diets can benefit from the fermentative method as it provides a cruelty‐free substitute for conventional animal‐derived vitamin B12 sources (Serin & Arslan, 2019). The microbial synthesis of vitamin B_12_ is consistent with the increasing inclination toward sustainable and ethical food production methods. The convergence of scientific advancements and sustainable practices is exemplified by the fermentative synthesis of vitamin B_12_ by *Propionibacterium shermanii* and *Pseudomonas denitrificans*, microorganisms that are integral to the era of precision nutrition (Fang et al., 2017). This novel methodology not only caters to the dietary requirements of individuals with varied preferences but also advances the overarching objective of establishing a food system that is more sustainable and resilient to environmental issues. The prime objective of this study is to clarify the metabolic pathway's effective synthesis of vitamin B12 via controlled fermentation. This study aims to investigate the potential health advantages that may be linked to vitamin B12 obtained through this fermentative method, with a particular focus on its capacity to rectify nutritional insufficiencies and enhance overall human health. The aim of this review is to provide significant contributions to the understanding of the nutritional effectiveness, sustainability, and feasibility of producing vitamin B12 using *Propionibacterium shermanii* and Pseudomonas denitrificans via microbes.

## 2 ROLE OF VITAMIN B_12_ IN REGULATING HEALTH

**FIGURE 1:**
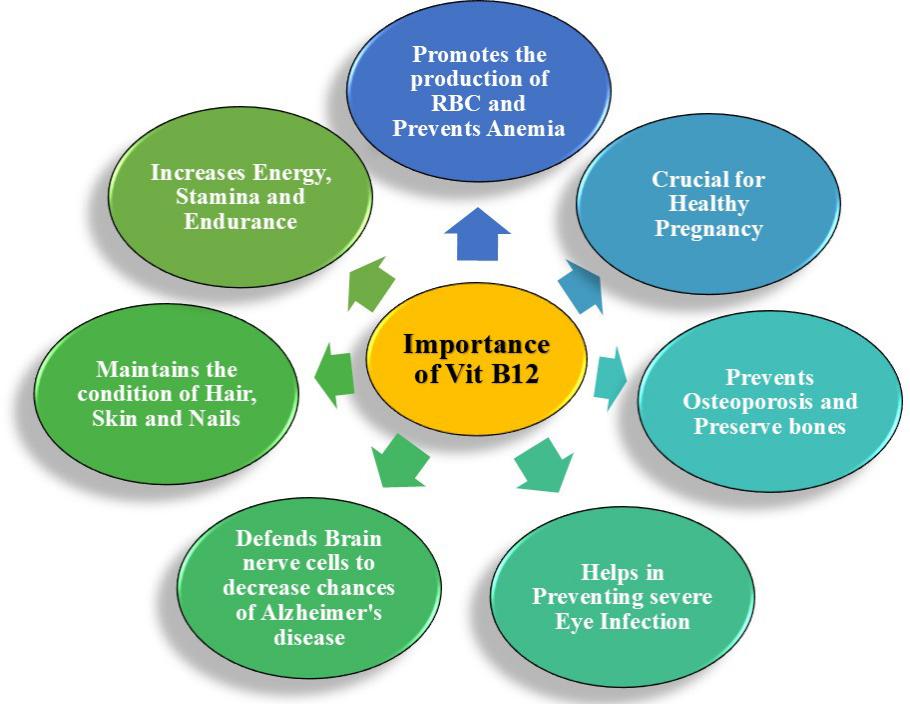
Pharmacological role of vitamin B_12_ in human body.

Meat, poultry, fish, eggs, and dairy foods contain cobalamin, a form of vitamin B_12_, organically. It could be incorporated into supplements or meals. Hematopoiesis and the formation of DNA require vitamin B_12_. It plays a significant function in the growth and operation of brain and nerve cells. The production of nucleotides and the metabolism of amino/fatty acids are biological activities that depend on vitamin B_12_ (Zhao et al., 2023). Lack of vitamin B_12_ negatively affects the production of phospholipids and the myelin layer in the neurological system by reducing methionine biosynthesis. Rodent peripheral nerve injury that has been demonstrated to be improved by vitamin B_12_ administration is assumed to be related to the inhibition of superoxide generation in neural tissue (Akbari et al., 2023). When vitamin B_12_ is used in conjunction with COX‐1 and COX‐2 inhibiting agents, the previously reported neuroprotective effect has been proven to be more powerful (Martínez‐Iglesias et al., 2023; Rauf et al., 2022). Conversely, prior research with individual participants confirms that vitamin B_12_ intake has a positive impact on intellectual performance (Demirtas & Erdal, 2023; Prajjwal et al., 2023). Vitamin B_12_ plays a critical role in overall development. Figure 1 is reflecting the significant role of B_12_ in the human body in various aspects.

**FIGURE 2:**
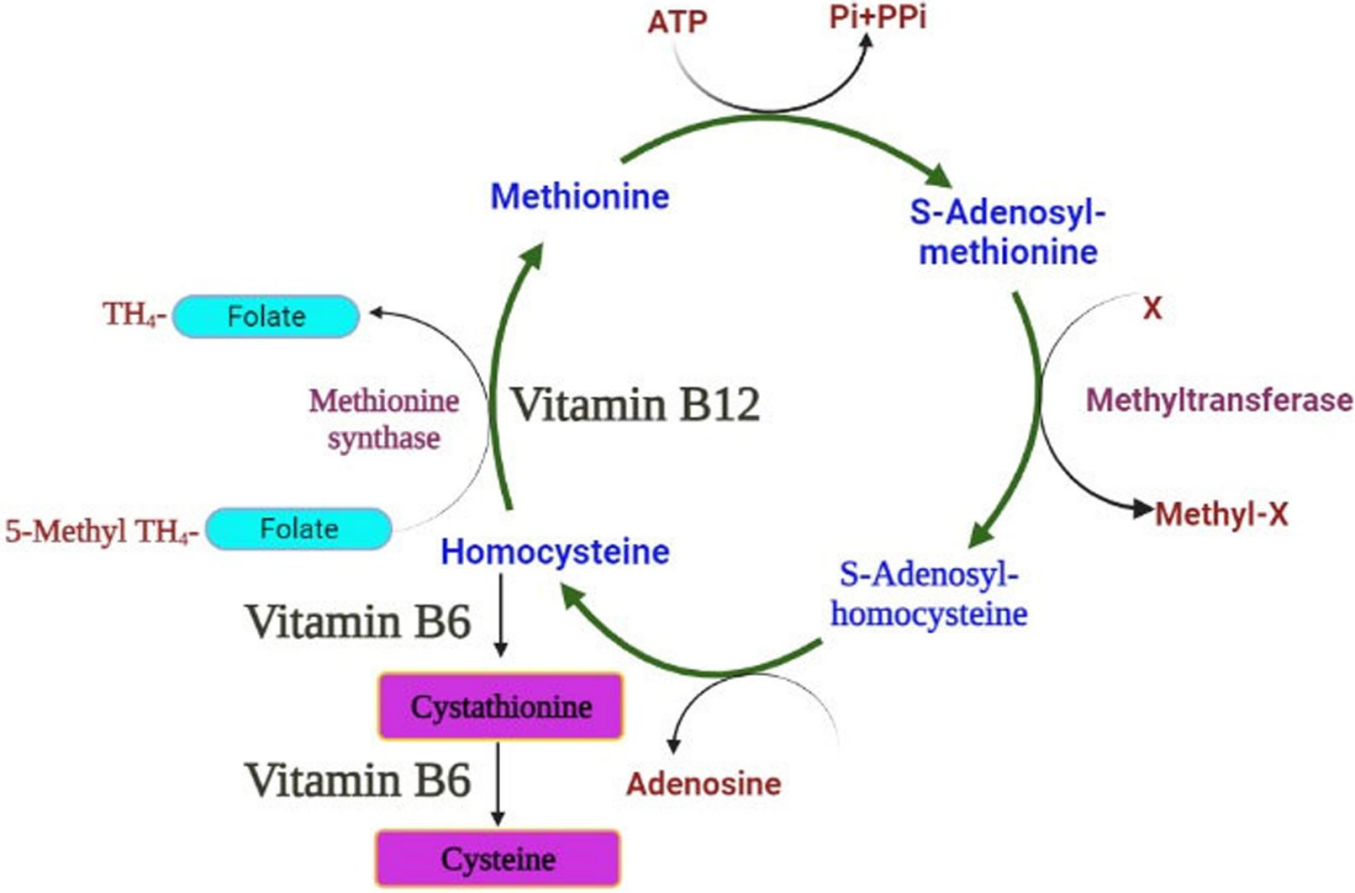
Metabolism of homocysteine with the help of vitamin B_12_.

Consuming functional foods that are enriched in vitamin B_12_ is a good approach to reduce human vitamin B_12_ insufficiency, especially among vegans. Since only bacteria and archaea can produce vitamin B_12_, its industrial manufacture is predominantly accomplished by microbial fermentation. Recent developments in metabolic engineering have made it possible to create microorganisms that are effective microbial cell factories to produce vitamin B_12_ by engineering them with powerful expression vectors, gene amplification, and promoters. Improvements to the fermentation process, such as substrate optimization, predecessor subjunction, precise oxygenation, and identification of growth‐limiting variables, have made it possible to implement tactics that will increase production (Kumar et al., 2023). Vitamin B_12_ contributes to the degradation of the protein homocysteine. Elevated levels of homocysteine have been linked to an increased risk of heart disease and stroke owing to its ability to promote the formation of blood clots, excess free radical cells, and to obstruct proper blood vessel function. If inadequate doses of vitamin B_12_ are consumed, homocysteine levels may increase. Even epidemiological studies have shown that taking vitamin B_12_ supplements can reduce homocysteine levels, but there is no conclusive evidence that this reduces the risk of cardiovascular events. Consequently, the American Heart Association opposes the frequent consumption of B complex supplements to reduce the risk of cardiovascular disease. Certain people with genetic variations that cause excessive homocysteine levels may benefit from vitamin B_12_ supplementation. Methionine synthase is a vitamin B‐dependent enzyme which uses the folate derivative S‐methyltetrahydrofolate (S‐methyl TH) as a methyl donor to catalyze the synthesis of methionine from homocysteine. Betaine is used as a methyl donor in a different route (not illustrated here) that is catalyzed by betaine homocysteine methyltransferase to remethylate homocysteine to methionine. Most biological methylation processes, particularly DNA methylation, call for the amino acid methionine in the form of S‐adenosylmethionine (Figure 2) (Tan et al., 2023).

Frequent investigations discovered a link between thyroid diseases (TD) and vitamin deficiencies. Antiparietal cell antibodies are a sign of a decreased capacity to absorb vitamin B_12_. Research published by Benites‐Zapata et al. (2023) investigated differences in vitamin B_12_ blood levels between sick people with and without tardive dyskinesia (TD), the incidence of vitamin B_12_ deficiency in TD patients, and the presence of antiparietal cell antibodies in individuals with TD. Vitamin B_12_ concentrations were decreased in hypothyroid patients than in normal individuals. No appreciable correlations among vitamin B_12_ levels and hyperthyroidism, autoimmune thyroid disease, or subclinical hypothyroidism were witnessed. B complex has a fundamental role in maintaining the regular operation of the nervous system. Due to their importance for brain function, deficits in the B vitamins B_6_, B_1_, B_9_, and B_12_ have been associated with depression (Wu et al., 2022). They are preventative toward hypercysteinemia, which is linked to a higher incidence of mental disorders (Cordaro et al., 2021). Additionally, a worse response to antidepressants has been linked to low levels of vitamins B_9_ and B_12_ (Oudman, 2020). Contributing to the DNA amalgamation of oligodendrocytes that produce myelin as well as synthesizing of myelin itself are two distinct functions of vitamin B_12_ (Berkins et al., 2021). An enzyme called methionine synthase uses cobalamin as a cofactor to catalyze the addition of a methyl group to the homocysteine molecule. S‐adenosylmethionine (SAM), a precursor to methionine, has been produced. SAM is vital for the methylation processes necessary for the correct synthesis and/or metabolism of DNA, RNA, neurotransmitters, and membrane phospholipids as well as for the proper function of the myelin sheaths of nerve fibers (Avalos et al., 2023). Few researchers proposed ways through which omega‐3 fatty acids and vitamin B_12_ impact the way by which brain functions. B_12_ and omega‐3 fatty acid consumption and metabolism have diverse effects on brain function. Indirectly through heightened oxidative stress and decreased brain omega‐3 fatty acid levels, elevated homocysteine levels and altered epigenetic modification affect brain neurotrophins and neuro‐vascular operation that could increase the likelihood of neurological diseases and dementia (Rathod et al., 2016). B_12_ is found in meals and supplements that have been fortified, perhaps making them simpler to absorb. There are several vitamin B_12_ tablets available. Despite claims that various forms, such as sublingual medications, or fluids injected beneath the tongue to be absorbed via the tissues of the mouth, had better absorption than regular tablets, research has not demonstrated a significant difference (Forgie et al., 2023). The recommended dietary limitations for vitamin B_12_ are far exceeded by the quantities seen in supplements. However, because sufficient intrinsic components are also required for absorption, the absorption of even higher levels is not guaranteed. Doctors may advise muscle B_12_ injections in situations involving significant vitamin B_12_ lacking caused by an insufficient intrinsic factor (pernicious anemia) (Park et al., 2023). Various diseases caused by the deficiency of vitamin B_12_ at different phases of life are represented in Table 2.

**TABLE 2:** Various diseases caused by the deficiency of vitamin B_12_ at different phases of life.

Different phases of life	Age	Amount of vitamin B_12_ required (mcg/day)	Rich natural source	Disease caused by deficiency	References
Newborn	0–6 months	0.4	Breast Milk (Mother should contain vitamin B_12_‐rich diet)	Breakdown to thrive anemia, hypotonia, developmental delay, microcephaly, and cerebral atrophy, lethargy, failure to thrive, vomiting, hypotonia, and arrest or regression of developmental skills	Dubaj et al. (2020), El Hasbaoui et al. (2021), Demirtas and Erdal (2023), Goraya and Kaur (2023)
Infant	7–12 months	0.5			
Toddlers	1–2 years	0.9	Almond milk	Anemia was found in 83%, developmental delay or regression, cerebral atrophy, neuroimaging, Hyperglycinuria	Agnoli et al. (2017), Tangeraas et al. (2023), Goraya and Kaur (2023), Kostecka et al. (2023)
Preschoolers	3–5 years		Unsweetened invigorated soya coconut or almond milk, oat		
Middle Childhood	6–8 years	1.2	Fortified breakfast cereal, Plain fortified soya yogurt	Neurological disorders, megaloblastic anemia and failure to thrive, elevated oxidative stress, hematological abnormalities	Charneca et al. (2023)
Children	Ears	1.8	Yogurt, plant‐based milk, low‐fat milk, fortified, cheese		Rowicka et al. (2023)
Young Teens	12–14 years	2.4	Shiitake Mushrooms, yeast, Tempeh, Nutritional, Yeast Spreads, Beetroot	Anemia in later life, nervous system damage, weight loss, fragile hair, knuckle pigmentation, hematologic (megaloblastic, macrocytic anemia), gastrointestinal (anorexia, glossitis), neurologic (demyelinization, paresthesia)	Umasanker et al. (2020), Tufan et al. (2012)
Adolescents	14–18 years		Swiss cheese Vitamin water, Butter squash, Whey Powder, Potato, Mushrooms, Noori, Tofu, Chickpea, Soybeans, low‐fat milk, low‐fat yogurt	Developmental delay, irritability, weakness, and failure to thrive	Henjum et al. (2023)
Adults	18+			Irritability, negativism, disorientation, confusion, agitation, amnesia, impaired concentration, insomnia, depression, psychosis, bipolar disorder, panic disorder, phobias and dementia	
Pregnancy	All ages	2.6	vitamin B_12_‐fortified breakfast cereals and bland soy drinks are also available. healthy yeast flakes with added vitamin B_12_ and yeast extracts, such as Marga	Neural tube defects in the baby, Infertility, Stomach cancer	Khosravi et al. (2023), Rooney et al. (2023), Bali and Naik (2023), Goraya and Kaur (2023)
Lactation period	All ages	2.8	Fortified Soy + Almond Milk, Marmite + Yeast Spreads, Nutritional Yeast, Fortified Cereals, Plant‐Based Meats, Cremini Mushrooms, Chlorella, Tempeh, Nori Seaweed, Seitan (wheat gluten)	Low birth weight, cognitive functions, gestational diabetes, Anemia	Behere et al. (2021), Goraya and Kaur (2023), Bali and Naik (2023)

## 3 PRODUCTION OF VITAMIN B_12_ FROM MICROBIOTA

Due to rising public awareness of health issues and the rising acceptance of nontraditional diets including vegan and vegetarian diets, the requirement of vitamin B_12_ among the food, nutraceutical, and beverage sectors has dramatically grown in the past few decades. Due to this, several attempts have been made over the years to optimize strain and procedures for synthesizing cyanocobalamin. Many researchers have been exploring the biosynthetic routes of organisms that produce cyanocobalamin because of the structural and organic discoveries of many vitamin B_12_ molecules in the 1970s. The complex structure of the molecule was subsequently determined to be the result of the “de novo” production of the molecule, which included more than 30 genes and multiple enzyme activities. Since no genetic proof exists that any eukaryotic creature can produce any isoform of cyanocobalamin, this route is limited to some bacteria and archaea. Industrial technologies and bacterial fermentation procedures can be used to produce vitamin B_12_ on a large scale. Today, the economically significant vitamin B_12_ is synthesized via *P. shermanii* and *P. denitrificans*, two modified strains of bacteria, which are used in the fermentation process. Up to 300 mg/L of vitamin B_12_ can be produced with this technique. This intricacy is a result of its complicated chemical production, which can take up to 70 steps (Acevedo‐Rocha et al., 2019). These microorganisms' ability to produce cobalamin beneath identifiable culture conditions (supplementation of precursors and metal ions, carbon/nitrogen sources, cultivation time, oxygenic/anoxygenic conditions, etc.) has been thoroughly studied. Their ability to synthesize cobalamin has been improved through spontaneous mutations (chemicals and ultraviolet light), genetic tampering (overexpression, alteration, parameter), and hereditary overexpression, transformation, and regulation (Bryant et al., 2020). The identified naturally occurring or recombinant bacteria that produce cobalamin include the genera *Acetobacterium*, *Methanobacillus*, *Aerobacter*, *Alcaligenes*, *Agrobacterium*, *Flavobacterium*, *Arthrobacter*, *Bacillus*, *Azotobacter*, *Escherichia*, *Corynebacterium*, *Propionibacterium*, *Eubacterium*, *Mycobacterium*, *Proteus*, *Methanosarcina*, and *Clostridium* (Rodionov et al., 2019; Shelton et al., 2019). In addition to mutation and overexpression of the biosynthetic genes and/or riboswitch sequences in the selected strains, the catalytic initiator of 5‐aminolevulinic acid (ALA), DMB, and cobalt ions were all introduced to enhance cobalamin production (Nguyen‐Vo et al., 2018).

**FIGURE 3:**
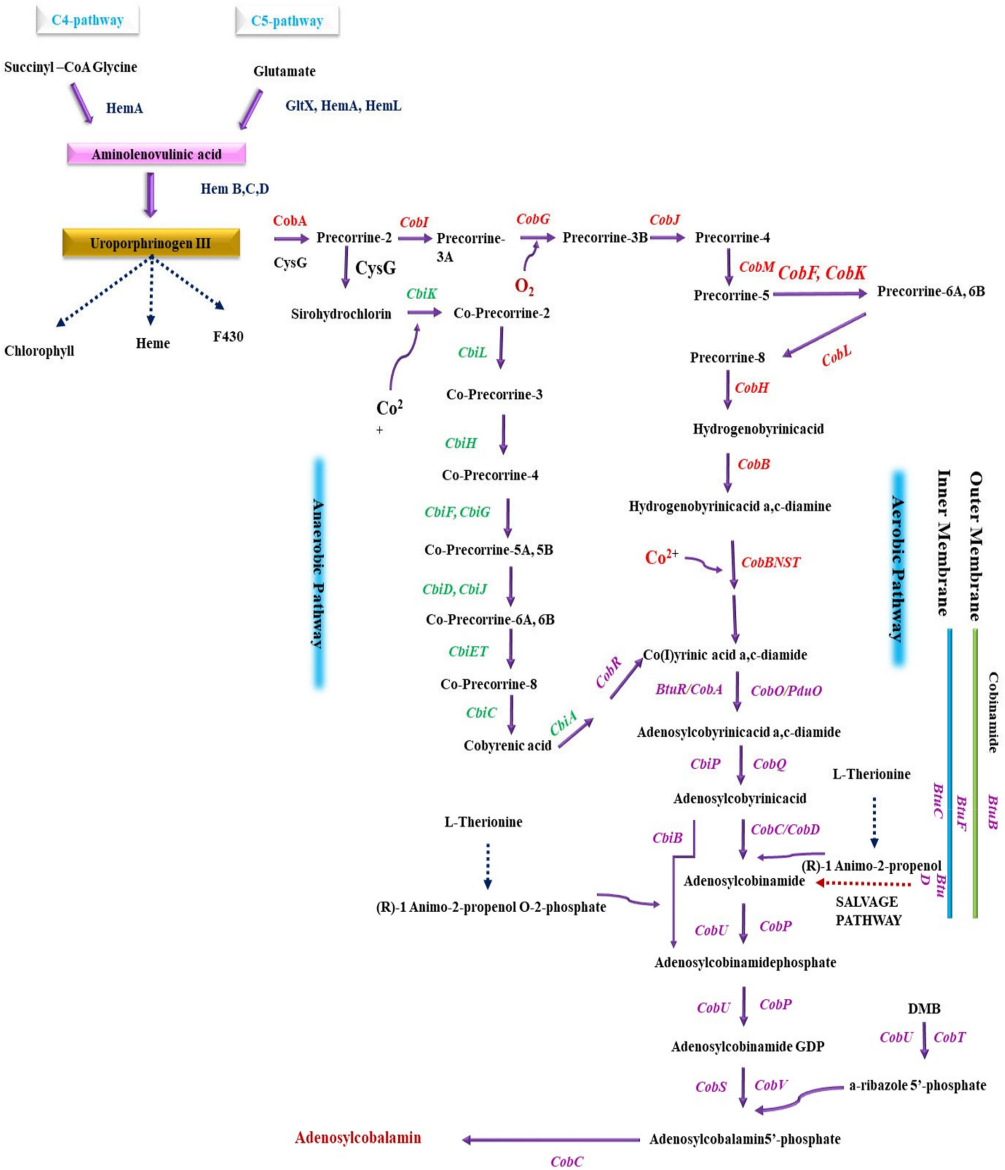
Diagram illustrating the salvage, anaerobic, and aerobic cobalamin biosynthetic pathways: The diagram commences with the aerobic pathway, which is illustrated for *P. denitrificans* or *Sinorhizobium meliloti* and the anaerobic pathway for the extensively researched bacteria *S. typhimurium* and *P. shermanii*.

The family of cobalamin, which also includes the vitamin B_12_ component cyanocobalamin, is made up of a corrinoid ring, an upper and a lower ligand. The top ligand may be adenosine, methyl, hydroxy, or a cyano group. Vitamin B_12_, which is produced by prokaryotes, shields mammals from pernicious anemia (Fang et al., 2017). Cobalamin can be synthesized by microorganisms through salvage or de novo (aerobic/anaerobic) pathways, which may each require as many as 30 enzymatic steps (Bryant et al., 2020; Lu et al., 2020). The de novo pathways consist of three major stages: (1) uroporphyrinogen III (Uro III) formation, the initial macrocyclic intermediate in tetrapyrrole synthesis; (2) cobinamide (Cbi) formation from Uro III, which involves the adenylation and formation of the corrin ring; and (3) nucleotide loop assembly, which entails the synthesis of a lower axial ligand, typically 5,6‐dimethylbenzimidazole (DMB), followed by its attachment to the corrin ring (Balabanova et al., 2021). The designations for the genes/enzymes involved in the oxygen‐dependent and oxygen‐independent pathways are, respectively, *cob*/Cob and *cbi*/Cbi. Key distinctions between these two pathways include the need for molecular oxygen to aid in ring contraction and cobalt insertion: in the aerobic pathway, this occurs only after the synthesis of hydrogenobyrinic acid *a*, *c*‐diamide (late stage), whereas in the anaerobic pathway (as shown in Figure 3), it occurs at the precorrin‐2 stage (early insertion). Additionally, in the anaerobic pathway, cobalt insertion occurs after the biosynthesis of a lower ligand, typically DMB (Danchin & Braham, 2017).

From aminolevulinic (ALA), tetrapyrrole compounds such as cobalamin, heme, and bacteriochlorophyll are created. Several bacterial species have complicated reliant and collaborative relationships with these tetrapyrrole compounds (Fang et al., 2017). A cobalamin riboswitch in the 5′ untranslated regions (UTR) of the relevant gene's controls vitamin B_12_ production and transportation to keep concentrations steady. Through microbial fermentation, vitamin B_12_ is produced on a large scale in the industrial setting, primarily using *Sinorhizobium meliloti*. *Propionibacterium shermanii*, or *Pseudomonas denitrificans*, these strains do have certain drawbacks, though, including lengthy fermentation cycles, demanding costly medium, and a dearth of acceptable inherent systems for strain building (Li et al., 2020). There have been few studies on metabolic engineering, and most studies carried out by these have concentrated on conventional techniques such as random mutagenesis and improving fermentation processes (Ma et al., 2017). *Escherichia coli* has recently attracted the curiosity of researchers as a model for the creation of vitamin B_12_. *E. coli* has developed into a well‐researched cell industry that is frequently utilized to produce a variety of compounds, including terpenoids, artificial alcohols, and poly‐(lactate‐co‐glycolate) (Fang et al., 2018). To enhance the synthesis of these chemicals, metabolic engineering and techniques from synthetic biology have also been widely used. In addition to producing ALA through the C4 and C5 routes, *Escherichia coli* is being employed as a microbial cell factory.

*Escherichia coli* can also produce vitamin B_12_ by salvage pathway. *Salmonella typhimurium*, a very similar strain, has the capacity to produce vitamin B_12_ from scratch. Numerous genes responsible for producing vitamin B_12_ in *S. typhimurium* have been demonstrated to activate in *E. coli* (Choi et al., 2016). The manufacture of vitamin B_12_ in *E. coli* was made possible by the introduction of 20 genes of the *S. Typhimurium* cob locus. These benefits make it easier for *E. coli* to produce vitamin B_12_ from scratch. Multiple research projects on vitamin B_12_ production have been carried out by multiple teams due to the intricacy of the route and its control of metabolism (Fang et al., 2017). List of enzymes involved in the production of vitamin B_12_ and their action mechanism have been shown in Table 3.

**TABLE 3:** List of enzymes involved in the production of vitamin B_12_ and their action mechanism.

Enzymes involved in the production of vitamin B_12_	Cofactor	Mechanism of action	References
Cobalt chelatase	ATP, Mg2+, S‐adenosylmethionine (SAM)	Catalyzes the insertion of cobalt into the corrin ring structure during vitamin B_12_ biosynthesis	Osman et al. (2021)
Cobalt‐precorrin‐6A reductase	NADPH	Reduces cobalt‐precorrin‐6A to cobalt‐precorrin‐6B, contributing to the formation of the corrin ring	Moore et al. (2013)
Cobalt‐precorrin‐5B C (1)‐methyltransferase	S‐adenosylmethionine (SAM)	Catalyzes the methylation of cobalt‐precorrin‐5B to cobalt‐precorrin‐6B, adding a methyl group to the corrin ring structure	Moore et al. (2013)
Cobalt‐precorrin‐4 methyltransferase	S‐adenosylmethionine (SAM)	Catalyzes the methylation of cobalt‐precorrin‐4 to cobalt‐precorrin‐5, another important step in vitamin B_12_ synthesis	Abdelraheem et al. (2022)
Adenosylcobalamin synthase	ATP, cob(I)alamin, S‐adenosylmethionine (SAM)	Converts cobinamide to adenosylcobalamin, the biologically active form of vitamin B_12_, through a series of reactions involving ATP and SAM	Balabanova et al. (2021)

## 4 FERMENTATION BY *PSEUDOMONAS DENITRIFICANS* FOR SYNTHESIS OF CYANOCOBALAMIN

### 4.1 Characteristics *of Pseudomonas denitrificans*

*Pseudomonas denitrificans* are distinguished by their rod‐shaped morphology, polar flagella, Gram‐negative cell wall composition, and aerobic metabolism. This bacterium has the potential to grow heterotrophically and can make vitamin B_12_ (Calvillo et al., 2022). One of the few bacteria which can synthesize vitamin B_12_ in an aerobic environment is *P. denitrificans*. *P. denitrificans*, as its name implies, is also able to undergo denitrification, a step in the nitrogen cycle when nitrate is converted into nitrogen gas (N_2_) (Balabanova et al., 2021). Considering the vast amount of information that is already discussed about *P. denitrificans*, still there is a great deal that is unexplained. Its cytochrome cc’ protein, which is present in the mitochondria and is crucial for the electron transport chain, has not yet undergone extensive research to determine how it relates to proteins that exist in photosynthetic bacteria conditions (Xia, Chen, et al., 2015; Xia, Peng, et al., 2015). Since the route is believed to have evolved to support fermentation activities, although this has not yet been convincingly established, the evolutionary suggestions of preserved genes encoding for vitamin B_12_ synthesis may give an understanding of the origins of metabolism. Concerning its commercial utility to produce vitamin B_12_, possible nitrate toxicity, or wastewater treatment applications, *P. denitrificans*' medicinal and ecological relevance is likewise unknown (Zhou et al., 2014). Since *P. denitrificans* is thought to be chronologically old, it offers a chance to investigate the evolution of metabolism. Its addition of denitrification to oxidative phosphorylation sheds light on the way it occupies and retains a niche despite changing N and O levels in the surroundings. *P. denitrificans* could be regulated to create other industrial substances, such as 3‐hydroxypropionic acid. Its capacity for denitrification has significant potential for handling wastewater. *P. denitrificans* has the potential to opportunistically cause meningitis in people. Fish intestines can additionally be infected by *P. denitrificans* (Xia, Chen, et al., 2015; Xia, Peng, et al., 2015).

The single circular chromosome of *P. denitrificans* ATCC 13867 has a genomic size of 5,696,307 bps and a Guanine + Cytosine concentration of 65.2%. Its genome has 2567 operons and 5059 protein‐coding genes, and 59.56% of its proteins are cytoplasmic while 19.41% are not. The remaining percentage is yet undetermined (Ainala et al., 2013). Its genome has 63 transfer RNAs and genes for each of the 20 amino acids. In addition, it has 816 transcription terminators and 1279 ribosome‐binding sites (Kumpangcum et al., 2023). The genome of *P. denitrificans* additionally contains genes that code for 26 enzymes essential to produce vitamin B_12_, and these genes are organized into two distinct chromosomal clusters. The first and second clusters contain genes associated with the fabrication of vitamin B_12_ (Tran et al., 2017). China, which includes the North China Pharmaceutical Company, the Henan Luyuan Pharmaceutical Company, the Hebei Yuxing Bio‐Engineering Company, and the Chinese CSPC Huarong Pharmacy Company, is the world's largest manufacturer of vitamin B_12_. These four companies are stated to have produced 31.41 tons of vitamin B_12_ in 2020, with a reported marketplace value of USD 339.8 million. Due to various studies from Huarong Pharmaceutical Company, it is believed that the strains utilized in their commercial productions are aerobic strains, and the most recent trademarks on bioprocess optimization with *P. denitrificans* exhibited in China claim volumetric manufacturing of up to 281 mg/L (Sun et al., 2023).

Improvements in culture medium optimization and bioprocessing parameters have also been made to increase vitamin B_12_ productivity in *P. denitrificans* in addition to genetic alterations. Tests have been conducted, for instance, on the impact of residue elements in media, dissolved oxygen management, pH, and the accumulation of various supplements. In this regard, it has been discovered that the synthesis of ALA and PBG, two of the main precursors of cobalamin, is greatly improved by the presence of Zn^2+^, whilst the addition of Co^2+^ and DMBI (dimethylbenzimidazole), the base inserted into the nucleotide loop, favorably impacts synthesis (Wang et al., 2021). Cobalamin synthesis increased by 13% because of the planning of experiments' optimization of the beginning concentrations of these three chemicals. The pH stability of cultures was impacted by the media composition, which also had a big impact on vitamin synthesis. A diet that uses glucose as a carbon source and betaine as a methyl donor was discovered to be beneficial for vitamin production while being employed in 120 m3 bioreactor setups (Wang et al., 2014). Furthermore, even while it is well established that betaine aids in the creation of various significant mediates, including glutamate, ALA, methionine, and glycine, it may also limit cell development at high concentrations. This is because betaine works as a methyl donor for the manufacture of vitamin B_12_ (Lyon et al., 2020; Pesqueda‐Cendejas et al., 2023). In *P. denitrificans*, oxygen transfer rate (OTR) optimization has received significant attention. Higher OTRs at the beginning of the culture process promote cell proliferation, but decreased OTRs at subsequent phases are essential for greater productivity (García‐Cabrera et al., 2020). Later research found that the enhanced production seen under poor oxygenation circumstances may be due to changes in cell shape that promote the transition from the cell growing phase to an extension stage which exhibits greater vitamin B_12_ synthesis (Lyon et al., 2020). Various multistep liquefied oxygen management techniques were created in response to this, where oxygenation and stirring were progressively decreased until thawed oxygen values plunged below 2%, resulting in a rise in output of about 17% (kazadi Mbamba et al., 2019). Despite having a negative effect on the development of cells, the adjunct of respiratory chain inhibitors, like as rotenone, might improve vitamin synthesis (Cheng et al., 2014). Finally, other sources of nitrogen and carbon are being investigated as cheaper replacements for more pricey improved glucose and sucrose including maltose syrup, glucose, steep liquor, beetroot molasses, and maize. A few of these substances may have a detrimental impact on pH stability and thus, the result of vitamin synthesis is also affected (Hernández‐Pérez et al., 2020). However, a viable and less expensive substitute for the conventional media formulations has been described using a mixture of maltose syrup, maize steep liquor, and betaine (Xia, Chen, et al., 2015; Xia, Peng, et al., 2015).

### 4.2 Synthesis of vitamin B_12_ by *Pseudomonas denitrificans*

It is feasible to exclude *Pseudomonas denitrificans* from surroundings like soil or water. As part of the separation procedure, samples are collected, diluted, and plated on the appropriate medium for development. Conventional microbiological methods, such as nutritional agar or broth enriched with the right nutrients for growth, can be used to cultivate *Pseudomonas denitrificans*. To encourage bacterial development, the culture is kept at the right temperature and pH. Optimizing nutritive circumstances is necessary for *Pseudomonas denitrificans* culture for vitamin B_12_ synthesis. Carbon sources (such as glucose or glycerol), nitrogen sources (such as ammonium salts or amino acids), and trace elements are examples of important nutrients. To produce vitamin B_12_, cobalt, and other necessary elements is very crucial. The appropriate quantities of these elements for optimum synthesis of vitamin B_12_ are able to be found through nutrient optimization studies. The procedure of fermentation is the phase that follows the bacteria that has been cultivated. A batch, fed batch, or continuous form of fermentation can be used (Balabanova et al., 2021). The fermentation medium is made to offer the best circumstances for bacterial development and the production of vitamin B_12_. To increase production, variables including temperature, pH, oxygen availability, and agitation are carefully regulated. To give the bacteria a chance to thrive and produce vitamin B_12_, the procedure of fermentation usually takes a few weeks. A complicated biochemical process is used by *Pseudomonas denitrificans* to produce vitamin B_12_. The route is made up of several successive enzyme processes. Precorrin‐3B synthase, precorrin‐4 methyltransferase, and cobalt chelatase are the main enzymes in this process. These digestive enzymes facilitate chemical processes that result in the production of vitamin B_12_. For effective vitamin B_12_ synthesis, the manifestation and operation of these enzymes must be controlled (Binod et al., 2010). Several methods can be used to strengthen the synthesis of vitamin B_12_. This entails tweaking fermentation settings, modifying Pseudomonas dentifrices strains genetically, and incorporating precursor chemicals or other precursors into the fermentation media. By altering variables such as temperature, pH, and dissolved oxygen levels, fermentation settings may be optimized to produce a setting that promotes the production of vitamin B_12_. The bacterium's metabolic pathways can be modified using methods involving genetic engineering to increase its capacity to manufacture vitamin B_12_.

**FIGURE 4:**
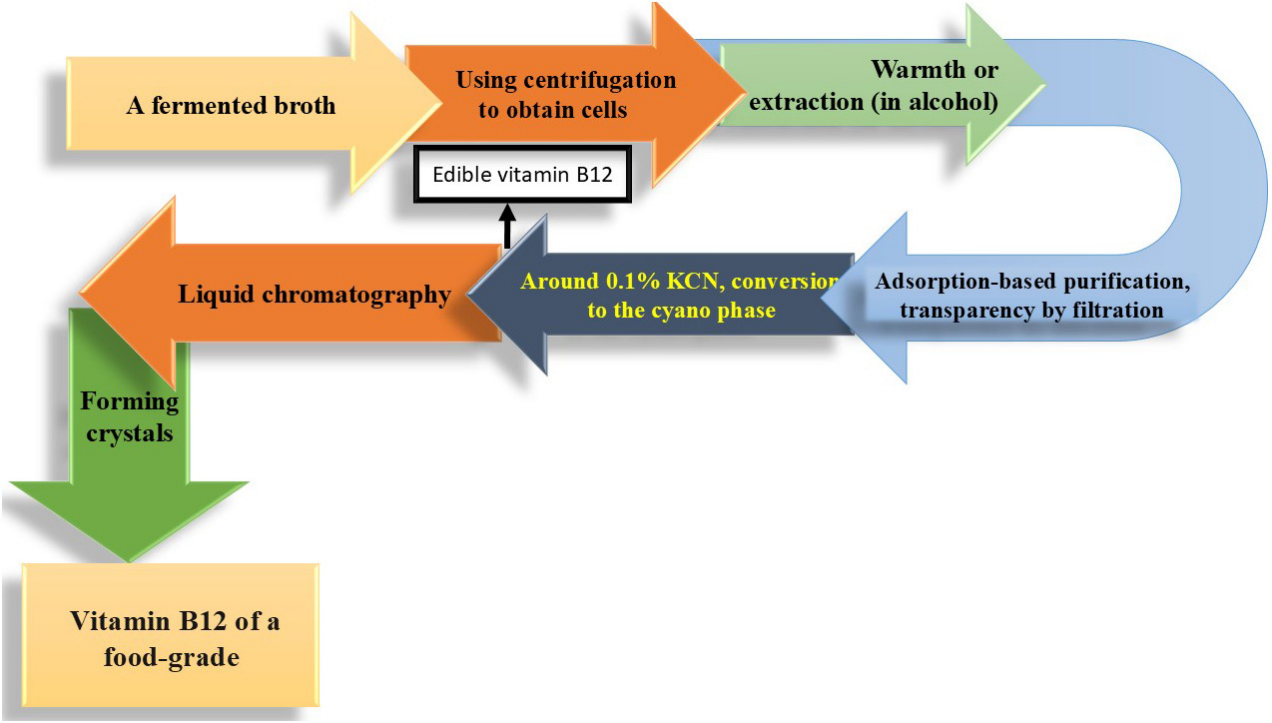
Downstream processing for the production of vitamin B_12_.

For instance, enhancing the biosynthetic pathway's essential enzyme expression can boost vitamin B_12_ production. Additionally, the required components for vitamin B_12_ biosynthesis can be supplemented to the fermentation medium in the form of precursor molecules or predecessors, thereby boosting production (Fang et al., 2017). The broth holding vitamin B_12_ must go through downstream processing after fermentation to separate and refine the desired component (Figure 4). Cell segregation, filtering, centrifugation, and chromatography methods are frequently used in the procedure. These procedures assist in removing undesirable cellular debris and contaminants, producing a vitamin B_12_ product that has been refined. The vitamin B_12_ can then be dried and made into different forms after being cleaned. It is crucial to keep an eye on and manage the vitamin B_12_ product's integrity throughout the course of production. This entails measuring and determining the existence of vitamin A using methods of analysis such as mass spectrometry and high‐performance liquid chromatography (HPLC) (Żandarek et al., 2023).

## 5 FERMENTATION BY *PROPIONIBACTERIUM SHERMANII* FOR SYNTHESIS OF CYANOCOBALAMIN

### 5.1 Characteristics of *Propionibacterium shermanii*

Propionibacterium spp. are Gram‐positive bacilli, which means they are catalase positive, have a length of 1–5 μm, are nonmotile, and do not produce bacterial spores. They are categorized as anaerobic or partially anaerobic bacteria. When present in anaerobic settings, PAB are very small and resemble cocci. These bacteria can exhibit pleomorphism in the presence of oxygen, taking on club‐shaped, V‐shaped, or Y‐shaped shapes. *Pseudomonas denitrificans* has an accelerated growth rate even in the presence of high salt concentrations, up to 6.5% NaCl, and performs best at a pH of 7.0 (with a range of 4.5–8.0). These bacteria are famous for their capacity to synthesize both propionic acid and vitamin B_12_. Based on its habitat, the Propionibacterium genus is divided into two separate groups: one group includes skin‐dwelling (acnes) bacteria, while the other group includes classical (dairy) bacteria. The first group includes species like *Propionibacterium lymphophilum*, *Propionibacterium acnes*, *Propionibacterium avidum*, *Propionibacterium propionicum*, and *Propionibacterium granulosum* (all of which are pathogenic microorganisms) that are found on human skin as well as in the gastrointestinal mucosa and oral. The second phylogenetic group includes the classical strains of bacteria. The first phylogenetic group consists of bacteria from the *Propionibacterium jensenii*, *Propionibacterium acidipropionici*, and *Propionibacterium thoenii* species. The next group of bacteria is made up of subspecies of *Propionibacterium freudenreichii* (subsp. *Freudenreichii* and subsp. *shermanii*). These subtypes differ in two characteristics: the capacity to decrease nitrates and the capacity to metabolize lactose. Bacterial strains generated from *P. freudenreichii* subsp. *freudenreichii* may reduce nitrates but cannot ferment lactose. Although *P. freudenreichii* subsp. *shermanii* strains include genes for the enzyme‐D galactosidase (EC 3.2.1.23), they are unable to reduce nitrates. As a gram‐positive bacterium, *Propionibacterium shermanii* preserves the crystal violet stain during the Gramme staining process. Its cell wall has a thick coating of peptidoglycan that accounts for this property (Dank et al., 2023). The bacteria are pleomorphic, which implies that it may have many sizes and forms. Depending on the growing circumstances, it can take the shape of rods, cocci, or even filamentous forms. The bacterium *Propionibacterium shermanii* is not mobile. It lacks flagella, which are bacterial motility‐causing extensions. *Propionibacterium shermanii* is predominantly anaerobic, indicating that it can grow without oxygen. It is also facultatively anaerobic. As a facultatively anaerobic organism, it can withstand and thrive in circumstances of oxygen shortages (Dank et al., 2022). Propionic acid production is one of *Propionibacterium shermanii's* distinguishing characteristics. A variety of carbohydrates, including lactate, glucose, and lactose, are fermented by this bacterium to create propionic acid, carbon dioxide, and trace quantities of acetic acid. *Propionibacterium shermanii* is essential to the dairy business. It serves as a beginning culture for the cheeses Emmental and the Swiss kind. The bacteria convert lactate into propionic acid during the cheese‐making process, giving these cheeses their distinctive flavor and texture (Blasco et al., 2011). *Propionibacterium shermanii* can produce vitamin B_12_, commonly referred to as cobalamin. The biological activities that take place in both humans and animals depend on this vitamin. The bacteria use a convoluted metabolic route to produce vitamin B_12_ (Calvillo et al., 2023).

Propionic acid bacteria may create vital molecules like vitamin B_12_, which can be utilized to create foods for vegan diets that are vitamin B_12_ enhanced. Trehalose and other vitamins from the B group are abundant in PAB feedstock. All traditional Propionibacterium genus bacteria are capable of fermentation. Cheese (Swiss‐style Dutch cheeses and vaccine components for Swiss cheeses), pickles, probiotics, and silage are all made with propionic acid bacteria (PAB). Preservation agents are made from PAB‐derived metabolites. Propionibacterium spp. may be found on herbaceous plants, in bovine rumens, in herbivore dung, soil, sewage, sludge, milk, pickles, water used to make oil, and fermented fruit fluids (Piwowarek et al., 2018). By encouraging the expansion of *Bifidobacterium* bacteria and shielding the animal from possible infections via the synthesis of bacteriocins, the species *P. freudenreichii* controls the intestinal microbiota. Along with an excellent source of trehalose and the vitamins B12, B9, and K, PAB also boosts the immune system and removes mycotoxins from the digestive tract. It has been demonstrated that adding PAB to the diet promotes both the usage of the substance and the physical progression of young animals (Singh & Vyas, 2022). Corresponding to Cousin et al. (2016), *P. freudenreichii* subsp. *shermanii* causes colon cancer cells to undergo apoptosis by producing propionate and acetate. In 2017, findings from studies on the use of *P. freudenreichii* subsp. *shermanii* as a beneficial component in Feta‐style cheese were published. Up to 7 days into the maturation process, Propionibacterium colony‐forming units (CFUs) developed in the finished product, and after 60 days, a propionic acid concentration of 52.1 mM was attained. In addition to adding flavor, the resulting Feta‐type cheese was distinguished by health‐indorsing qualities and prolonged the expiry day. A good source of vitamin B_12_, feed (Bioprofit™) is generated as well using PAB strains because they help animals assimilate iron and calcium and guard the finished product against fungus. Certain PAB strains are fed to animals as probiotics (Angelopoulou et al., 2017). By encouraging the evolution of *Bifidobacterium* bacteria and defending the body against the growth of harmful microorganisms through the production of bacteriocins, *P. freudenreichii* controls the flora of the intestines. PAB can remove mycotoxins from the gastrointestinal system. They produce trehalose, folic acid, vitamins B_12_, and H, as well as encourage the immune system and reduce the carcinogenic realizes of fecal enzymes. When PAB is added to the meal, its usage increases, promoting the development of young animals (Piwowarek et al., 2018). T82 strain's genome also included genes that could be related to glycogen metabolism. The *P. freudenreichii* ssp. *shermanii* CIRM‐BIA1T strain also possessed this trait (Falentin et al., 2010). The series encoding glycogen synthase (EC 2.4.1.21), glycogen phosphorylase (EC 2.4.1.1), and branching glycogen enzymes (EC 2.4.1.18) are necessary for the T82 strain to be able to synthesize this chemical. *P. acnes* also possessed several of these genes. These enzymes are anticipated to be responsible for intracellular glycogen buildup and/or hydrolysis because *P. freudenreichii* and *P. acnes* cannot ferment extracellular glycogen (Piwowarek et al., 2020).

### 5.2 Synthesis of vitamin B_12_ via *Propionibacterium shermanii*

Cobalamin is produced industrially only by fermentation procedures using microorganisms, mainly *P. denitrificans*, because of its intricate nature (about 70 steps) and the high expense of its chemical synthesis. Food items are increasingly being enhanced with vitamin B_12_ by in situ fermentation in recent years. The Wood‐Werkman route is used by *P. freudenreichii* varieties, which are Gram‐positive rod‐shaped bacteria that can synthesize huge amounts of propionic acid. *P. freudenreichii*, in comparison to aerobic generators of vitamin B_12_, has the benefit of having received the FDA's GRAS designation and the EFSA's Qualified Presumption of Safety (QPS) designation. It is important to emphasize in this regard that *P. freudenreichii* is the only microbe having GRAS certification that can synthesize the physiologically active form of vitamin B_12_. Due to this distinguishing characteristic, *P. freudenreichii* plays a special role in the industrial synthesis of vitamin B_12_ and its application to the microbiological replenishment of food and feed. These bacteria efficiently synthesize therapeutically useful vitamin B_12_, with just minor amounts of inert analog synthesis (Piwowarek et al., 2018).

**FIGURE 5:**
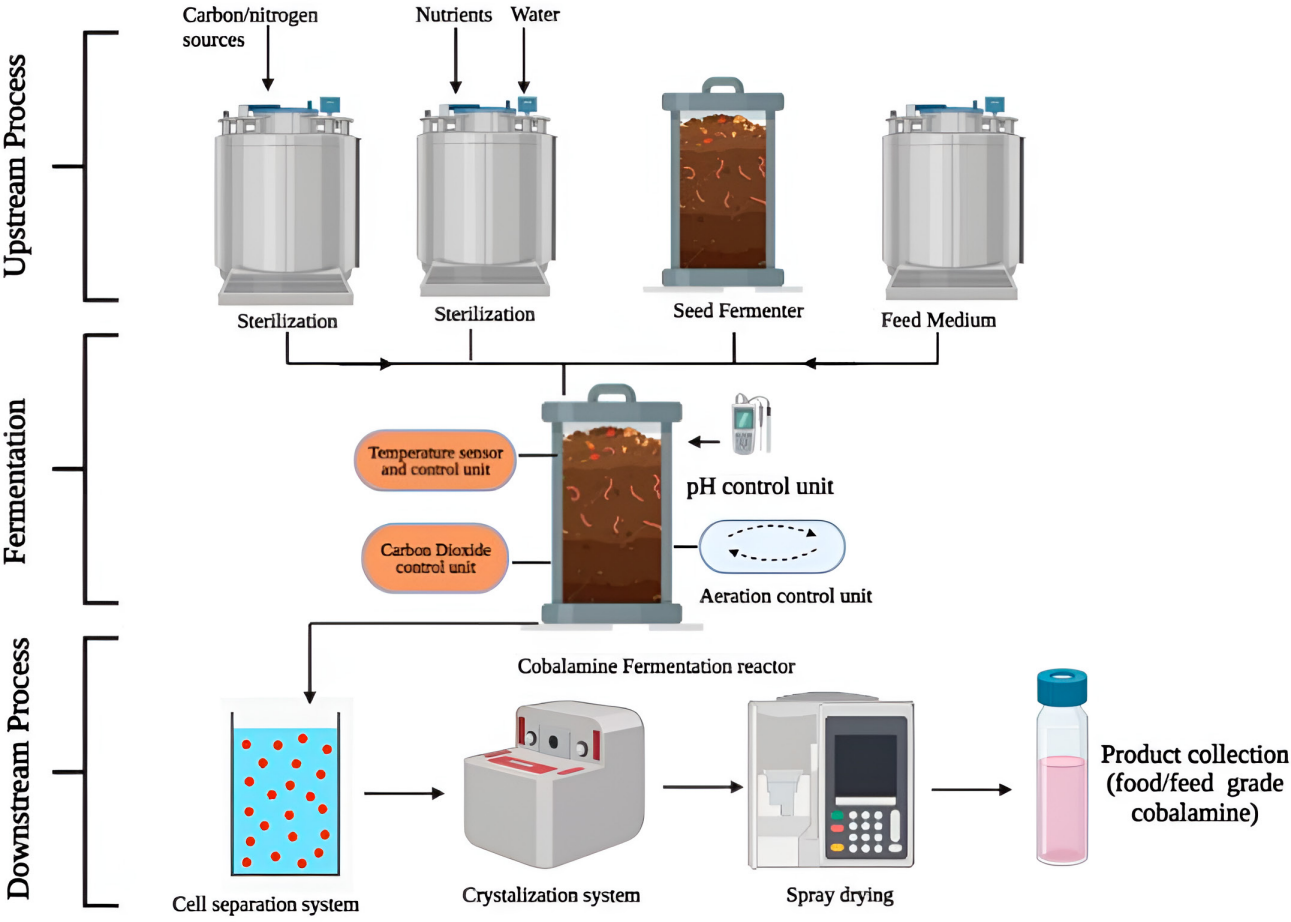
Exemplary commercial cobalamin manufacturing flowchart that applicable for *Propionibacterium shermanii*.

There are several known organisms that produce the B_12_ vitamin and are categorized as GRAS species, for a variety of reasons the PAB is preferred by the food and beverage sector. *Lactobacillaceae reuteri* bacteria can synthesize vitamin B_12_ which is ineffective for human consumption because they are incapable of adding ligands beyond the adenine in the bottom half of the ring (Watanabe & Bito, 2018). To strengthen the quantity of vitamin B_12_ in *P. freudenreichii*, various genetic engineering techniques were explored. For instance, it has been claimed that improving cobalamin biosynthesis involves using a genome‐shuffle method and overexpressing selected of the key genes implicated in cobalamin amalgamation. To get superior cobalamin growers, the primary commercial strains are frequently generated by accidental mutations utilizing a variety of mutagenic stimuli, such as ultraviolet light or chemical substances. These high‐yielding strains of *P. freudenreichii* often exhibit greater tolerance and resistance to propionic acid. Propionibacterium may synthesize vitamin B_12_ in two different methods. The first technique uses microbial cultures to add vitamin B_12_ to specific fermented food items (Van et al., 2011). To increase the quantity of vitamin B_12_ in *P. freudenreichii*, various genetic engineering techniques were explored. It has been claimed that improving cobalamin biosynthesis involves using a genome‐shuffle method and overexpressing specific key genes required in cobalamin production. To get superior cobalamin growers, the primary commercial strains are frequently generated by accidental mutations utilizing a variety of mutagenic stimuli, such as ultraviolet light or chemical substances. These high‐yielding strains of *P. freudenreichii* often exhibit greater tolerance and resistance to propionic acid. Propionibacterium may synthesize vitamin B_12_ in two different methods. The primary technique uses microbial cultures to add vitamin B_12_ to specific fermented food items (Van et al., 2011). *P. freudenreichii* are autonomous anaerobic strains that produce cobalamin through microbial biosynthesis. Oxygen is mandatory for the production of DMBI and its attachment to the corrin ring, even though they only produce high cobalamin outputs at extremely low oxygen concentrations (Deptula et al., 2017). A typical growing process is divided into two stages: a first stage in which the cells are cultured in complete anaerobic conditions, and a second stage—typically after 72–96 h of cultivation—in which soft oxygenation is generated by agitation to create the microaeration necessary for the formation of DMBI and cobalamin (Mauerhofer et al., 2019). By enabling its frank source in the development of food items, these producers of vitamin B_12_ were able to broaden the breadth of their market as depicted in Figure 5 (Dudko et al., 2023).

As a result, Deptula et al. (2017) investigation into the fortification of tempeh revealed that *P. freudenreichii* has been successfully used in in situ food fortification using food‐like environments such as cheese‐like propionic medium, whey‐based liquid medium, cereal matrices, and even in the fortification of tempeh. This method of fortifying food enables an increase in cobalamin content by utilizing unusual sources and obtaining the necessary daily consumption levels of vitamin B_12_ through small amounts of fermented goods. This approach works well despite having final cell densities that are considerably lower and reported production levels than with standard medium. Surprisingly, these microorganisms can produce large levels of propionic acid, which, over time, can become harmful and obstruct cell development, as shown in prior investigations. In the bioprocessing field, several optimization strategies have been investigated to address the development of propionic acid (Chamlagain et al., 2018). Results from using in situ product removal (ISPR) techniques to synthesize propionic acid and vitamin B_12_ together have been positive. It has been demonstrated that volumetric manufacture of vitamin B_12_ approximately 40 mg/L and 60 mg/L utilizing ISPR technologies is possible using expanded‐bed adsorption bioreactors (EBABs) and high biocompatibility resins, such as ZGA330. While desorption occurs in EBABs, propionic acid is maintained in the resin, allowing the culture to pass down the chromatographic column without clogging (Signorini et al., 2018). The concurrent enhancement of vitamin B_12_ and propionic acid synthesis has been studied under various culture conditions, carbon and nitrogen sources, and the inclusion of medium dietary supplements, which include DMBI (Wang et al., 2020). Both corn stalk hydroxylates and the mixture of glucose and glycerol were shown to be effective carbon sources in an EBAB system, with reported volumetric CNC cyanocobalamin manufacturing rates of 43.2 mg/L and 47.6 mg/L, correspondingly. Cofermenting *P. freudenreichii* with other microbes that may metabolize propionic acid is an intriguing method for lowering propionic acid content. For instance, co‐cultivating *Ralstonia eutropha* and *P. freudenreichii* increased cobalamin synthesis from 6.73 mg/L to over 19 mg/L. Cofermentation has been effectively used to create multiple products at once or to strengthen different cell cultures in addition to reducing propionic acid (Wang et al., 2020). The cocultivation of *Lactobacillaceae plantarum* and *P. freudenreichii* (now known as *Lactiplantibacillus plantarum*) allowed for the concurrent manufacturing of folate and vitamin B_12_, and a recent patent for the cofermentation of a Basidiomycota of a Basidiomycota strain with *P. freudenreichii* allowed for the concurrent fabrication of vitamins D and B_12_. For microbiological stability as well as security, *P. freudenreichii* and *Lactobacillaceae brevis* (now known as *Levilactobacillaceae brevis)* were cocultivated while producing vitamin B_12_ in situ in bread dough using the whey‐based medium (Zheng et al., 2020). One typical tactic for raising productivity is fortification with cobalamin precursors. It has frequently been said that the inclusion of common precursors and necessary substances, such as ALA and Co^2+^, is advantageous for vitamin synthesis. DMBI can be produced independently by all *P. freudenreichii* strains; however, its biosynthesis is not very high. Furthermore, since oxygen is obliged for the synthesis of DMBI, its creation is not achievable under purely anaerobic circumstances. The cells start to accumulate inactive forms of vitamin B_12_, such as cobinamide or pseudovitamin B_12_, if DMBI accessibility is reduced. Instead, the active form of vitamin B_12_ cannot generated. It has been repeatedly documented that adding DMBI or even DMBI precursors, such as riboflavin or nicotinamide, helps the formation of cobalamin (Chamlagain et al., 2016). The addition of vitamin B_12_ mimics has also been observed by other research teams to reduce feedback inhibition and boost cobalamin synthesis. *P. freudenreichii* cultures are intriguing in industrial settings due to their ability to grow in a variety of complex carbon and nitrogen sources as well as waste and spent media, such as molasses, crude glycerol, waste frying sunflower oil, tomato pomace, liquid acid protein residue of soybean, and vegetable juice spent media (Assis et al., 2020).

## 6 FUTURE PERSPECTIVES

The production of this essential mineral through fermentation by *Pseudomonas denitrificans* and *Propionibacterium shermanii* holds great promise as the demand for vitamin B_12_ supplements keeps rising due to a rise in its prevalence and increased awareness of its health benefits. To improve the effectiveness, sustainability, and applicability of this manufacturing technique, several future views and developments might be investigated. Future genetic engineering and metabolic engineering studies on *P. denitrificans* and *P. shermanii* may result in the creation of improved strains with greater vitamin B_12_ production. Future genetic engineering and metabolic engineering studies on *P. denitrificans* and *P. shermanii* may result in the creation of improved strains with greater vitamin B_12_ production. Researchers can develop robust strains that can produce more vitamin B_12_ during fermentation by specifically targeting genes involved in cellular processes and vitamin B_12_ production. The fermentation process will need to continue to be optimized to produce vitamin B_12_ at its highest potential. Improved fermentation conditions, such as oxygen availability, pH, temperature, and nutrient supplementation, can be the subject of research to help provide an environment that increases productivity and lowers production costs. Cobalt is now the main substrate for vitamin B_12_ synthesis; however, it can be costly and scarce. Alternative and more environmentally friendly substrate sources to produce vitamin B_12_ might be explored through research. The total production process may become more economically feasible and ecologically friendly by selecting cost‐efficient and environmentally suitable substrates. Fermentation‐based large‐scale manufacturing of vitamin B_12_ will require advancements in bioreactor technology and process scalability. In addition to meeting the growing demand for vitamin B_12_, efficient and economical industrial methods will help increase access to it for underserved populations, particularly in areas with little resources. Even though the potential health advantages of vitamin B_12_ are widely known, further investigation into its uses in a variety of industries, including medicine, animal husbandry, and agriculture, is possible in the future. Personalized medicine may have novel applications for vitamin B_12_ if its involvement in the prevention or treatment of illnesses is studied.

## 7 CONCLUSION

Through a complicated process, microbes produce vitamin B_12_, which is widely used in the food and pharmaceutical industries. Heme, cobalamin, and siroheme are examples of tetrapyrrole compounds. Rather than being antagonistic, these molecules interact and depend on one another with other substances in different types of bacteria. To maintain constant levels of vitamin B_12_, riboswitches regulate the transcriptional or translational level of vitamin B_12_ synthesis and transport. Researchers and numerous sectors of the food, feed, medical, and pharmaceutical industries are becoming interested in PAB due to its great potential. By using strains of bacteria such as *P. denitrificans* and *P. shermanii*, vitamin B_12_ is created by fermentation by microbial means. These bacteria have complex enzyme machinery that allows them to use a wide range of carbon sources. The ability of propionic acid bacteria to produce a range of physiologically valuable compounds from byproducts of industrial processes, such as propionic acid, vitamin B_12_, trehalose, and bacteriocins, is the most important fact about the usage of these bacteria for commercial reasons. Therefore, the biotechnological use of PAB may help to reduce environmental pollution by transforming the waste into valuable and useful components for other sectors. Further research is needed to increase the biosynthetic efficiency of these metabolites to scale up manufacturing, for example, by employing waste products, which may be less expensive than the current production procedures. It should be highlighted that the production of cyanocobalamin is now subpar and faces several challenges before reaching its full potential as a cost‐effective and successful industrial bioprocess. The underlying issue is that volumetric production levels are often only 200–300 mg/L, even in aerobic strains with higher production rates like *Pseudomonas denitrificans*. This is significantly less than the values obtained by similar fermentation techniques, such as those for vitamin B_12_. The long and costly fermentation cycles are also a result of the requirement for costly media components, such as large amounts of complicated nitrogen sources and supplements like betaine. Getting enough cobalt into the soup could potentially be difficult in terms of finances and environmental conditions. Further efforts in bioprocessing, downstream, and media components (with less expensive or recycled components) should be done to increase the ecological sustainability and economic viability of vitamin B_12_ biotechnological synthesis. The primary reasons for the low productivity of the producing strains that are now available are the inhibition of the cbi operon and cysG by the cobalamin riboswitch and other downregulating mechanisms. Genetic engineering might be required to overcome this constraint, which would not be well received by the public. Fruitarians and vegans are very concerned about the use of genetically modified organisms in their diets (Acevedo‐Rocha et al., 2019). The two main producers of vitamin B_12_ used in manufacturing operations are *P. freudenreichii* (206 mg/L) and *P. denitrificans* (214 mg/L). These bacteria grow slowly (they ferment for 180 h on average), making it challenging to genetically alter them. It is necessary to add more generating hosts to increase the current assembly of vitamin B_12_. With the aid of thorough knowledge of the steps involved in the biosynthesis of vitamin B_12_ and their guidelines, the de novo anabolism of the vitamin in well‐researched and commercially acceptable organisms such as *B. megaterium*, *E. coli*, and the soil species *S. meliloti* which can be replaced by strains generated at elevated yields has been engineered over the past 10 years. This has created significant engineering challenges for further study. Scientists are able to look into new concepts for improving B_12_ biosynthesis because of the usage of inexpensive medium components for fermentation, impacted gene changes, and an efficient explanation of designed strains for vitamin B_12_ synthesis for short periods of time (24–48 h). Only a few species *P. denitrificans*, *P. freudenreichii*, *R. capsulatus*, *S. meliloti*, *S. typhimurium*, *B. melitensis*, and *Rhodopseudomonas palustris* are known to use their recombinant advocates and riboswitches, even though the lists of microbiological and genetic resources are continually expanding. Using modern comparative genomics and metabolic restoration tools, it will be possible to identify potential hosts as well as genes, regulatory elements, or metabolic pathways for an additional efficient cobalamin metabolic rate or the relevant also simultaneous trials.

## AUTHOR CONTRIBUTIONS

**Anjali Tripathi:** Writing – original draft (lead). **Vinay Kumar Pandey:** Project administration (lead). **Parmjit S. Panesar:** Supervision (lead). **Anam Taufeeq:** Methodology (equal). **Hridyanshi Mishra:** Investigation (equal). **Sarvesh Rustagi:** Resources (equal). **Sumira Malik:** Data curation (equal). **Béla Kovács:** Writing – Review and editing (equal); Funding acquisition (equal). **Tejas Suthar:** Methodology (equal). **Ayaz Mukarram Shaikh:** Writing – Review and editing (equal); Funding acquisition (equal).

## FUNDING INFORMATION

Project No. TKP2021‐NKTA‐32 was implemented with support from the National Research, Development, and Innovation Fund of Hungary, financed under the TKP2021‐NKTA funding scheme, and supported by the University of Debrecen Program for Scientific Publication.

## CONFLICT OF INTEREST STATEMENT

The authors declare no conflict of interest.

## Data Availability

No data have been used in this article.
